# Factors influencing the inclusion of diverse volunteer patients within medical student primary care placements

**DOI:** 10.1111/medu.15562

**Published:** 2024-11-03

**Authors:** Mohammad Malik, Leanne Tyson, Pauline Bryant, Payal Patel, Richard Young, Joanna Semlyen

**Affiliations:** ^1^ Norwich Medical School University of East Anglia Norwich UK; ^2^ Milton Keynes University Hospital Milton Keynes UK

## Abstract

**Introduction:**

Research shows that medical students are graduating with inadequate teaching on diverse patients and insufficient experience of working with diverse patient groups. The inclusion of patients from diverse groups is necessary in healthcare teaching to ensure medical students are adequately prepared for practice. In this study, we explored the perspectives of General Practitioner (GP) tutors on the recruitment of diverse volunteer patients for medical student primary clinical care placements. In particular, we focused on the current representation of diverse volunteer patients, barriers affecting their inclusion and recommendations to help with this.

**Methods:**

Focus groups were carried out with GP tutors involved in the recruitment of volunteer patients from one region in the United Kingdom. Transcripts were analysed using Thematic Analysis.

**Results:**

Participants acknowledged the importance of ensuring that medical students have clinical experience in assessing and managing patients from diverse populations, but most did not actively think about the diversity of the patients they were recruiting. Instead, recruitment was driven by the need to cover the curriculum and teaching requirements.

To ensure that students' learning was not diminished and recognising time was a significant factor, participants automatically discounted certain patients from being a volunteer patient. They acknowledged that they did not feel comfortable identifying patients based on their demographics and were more likely to invite patients who had been volunteer patients before.

**Discussion:**

Suggested solutions to overcome the factors affecting the recruitment of diverse patients are presented. Patient populations will continue to become more diverse, and therefore, medical schools must prepare their students for this and encourage GP tutors to make a conscious effort to recruit diverse patient volunteers for teaching.

## Abstract 2

Diverse volunteer patients offer improved preparedness for practice in medical and healthcare trainees, leading Mohammad et al. to explore factors influencing their inclusion.

## 1 INTRODUCTION

Inclusive teaching practice is necessary in healthcare education to ensure medical students are adequately prepared for practice with a diverse population and have garnered relevant clinical practice and knowledge. The General Medical Council^1^ states that ‘Medical school curricula must give medical students experience in a range of specialties, in different settings, with the diversity of patient groups that they would see when working as a doctor. It is essential that medical students gain clinical experience working with patients from diverse groups to enable them to deliver excellent clinical care to all patients on qualification’ (page 33).

There is a growing awareness that medical students may not be getting the necessary experience of working with diverse patients, despite extensive medical training.^2^, ^3^ It is important for example, that medical students are educated on how dermatological conditions can present differently in persons of colour,^4^ and as such, can be missed, resulting in delayed presentation of melanoma in Black and Hispanic patients with consequent poorer outcomes compared with White patients.^5^ There are also differences in the manifestation of pain perceptions, with African American patients reporting greater pain and suffering when compared with White patients.^6^ Additionally, medical students have suggested that they do not receive adequate education regarding LGBTQ+ related medical education.^7^

Research has shown that medical students who spent >75% of their study time with students from different backgrounds or who participated in a greater number of diversity‐related extracurricular activities were more likely to rate themselves as prepared to care for diverse patients overall.^8^ Thuraisingham and colleagues^9^ reiterate the need to understand this perspective and use it to design learning activities for students. Furthermore, Rayan‐Gharra et al.^10^ noted that exposing students to community care and the impact of socio‐cultural factors on health actively inspired them to consider future careers in the community.

A central element of medical students' learning is attending clinical primary care placements at local General Practitioner (GP) surgeries. In the United Kingdom, primary care is the first point of contact in the healthcare system and the main way that people access the National Health Service (NHS). General practice is a critical part of the NHS, with GPs providing patients with continuous care—managing acute illness and long‐term conditions and providing preventative care. Students will attend these practices to receive curriculum‐orientated teaching from trained GPs (GP tutors) and have the opportunity to assess and examine ‘volunteer patients’ in order to gain clinical experience. These are intentionally recruited by GP tutors or administrative staff to provide an opportunity to discuss their relevant medical conditions and practice clinical examinations in a safe and controlled environment.

We know that students can obtain a wealth of experience in general practice through meeting patients with common chronic diseases.^11^ Considering that some diseases disproportionately affect patients from different marginalised groups, it is therefore essential that these patients are adequately represented in medical student clinical placements to ensure diversity of medical teaching.^12^

The UK's Medical Schools Council^13^ has made efforts to support diversity within medical school training and make it more inclusive by auditing teaching materials to ensure they cover diversity appropriately. One example of this is through the use of supplementary materials such as *Mind the GAP*,^14^ which is a clinical handbook of signs and symptoms of black and brown skin. However, studies still report that medical students and doctors report a lack of preparedness for diverse patient care.^15^, ^16^

At present, research exploring the representation of diverse patients within primary care clinical placements for medical students remains limited. Although Forrest and colleagues^12^ explored cultural diversity and inclusion in medical schools from the perspective of primary care educational leads, they did not specifically evaluate the representation of diverse volunteer patients during clinical placements and barriers affecting recruiting of these patients. Therefore, to explore this issue, we conducted a qualitative study involving GP practices offering clinical placements to medical students from *Norwich Medical School*. The aim of the study was to identify potential factors that could affect the inclusion of diverse volunteer patients in primary care clinical placements for medical student teaching. Due to the lack of ethnically diverse patients in the Norfolk area, we chose to specifically focus on the recruitment of ethnically diverse patients as an example of diverse recruitment. Because medical students are allocated job placements based on randomisation and also need to be comprehensively prepared to practice, it is essential that they have the necessary experiences working with ethnic minorities, as they may be allocated to a different region of the country that is more ethnically diverse. To our knowledge, the issue of ethnic minority patient representation in primary care clinical placements has not been explored, particularly from the perspective of GP tutors who recruit volunteer patients.

## 2 METHODS

### 2.1 Design

A qualitative focus group study was carried out between June 2021 and 2022 across multiple GP practices involved in medical student teaching and dealing with volunteer patients at Norwich Medical School. Focus groups were chosen to generate discussion and encourage group interaction. By facilitating open discussion, focus groups enable participants to raise issues of importance to them individually and explore them as a group. This approach uncovers shared meaning and experiences whilst also highlighting any concerns and priorities within the group. The study gained ethical approval from the University of East Anglia Faculty of Medicine and Health Sciences (FMH) Ethics Committee.

### 2.2 Participants and recruitment

At Norwich Medical School, medical students regularly attend university‐affiliated primary care placements. Students will attend these practices to receive curriculum‐orientated teaching from trained GP tutors, as well as the opportunity to gain clinical experience working with patients. The placements and tutors are selected and trained by the medical school for this role and are supported by comprehensive learning plans setting out the requirements of each teaching session. In preparation for these teaching sessions, the GP tutor and/or their administrative staff will identify appropriate patients for the medical students to and subsequently ask for their consent for their involvement. Patient recruitment may take place via the use of searches of clinical information systems for particular diagnostic codes or long‐term medication; by personal knowledge of patients by their GPs; occasionally opportunistically; or frequently a combination of the foregoing. Most patients are specifically invited purely for the purposes of interacting with students, but sometimes patients are already attending the practice for an appointment (and this is usually the case with opportunistic contacts). Students are then encouraged to speak to these patients to understand their medical history and examine them to practice identifying clinical signs in a controlled and supported environment. This exercise is usually followed by a discussion with the GP tutor to explore the case history, reflect on the experience and identify areas of learning.

For this study, emails were sent by MM to the GP tutors at the surgeries offering primary care placements to Norwich Medical School students. The email contained a Participant Information sheet and Consent form. Only those who completed and returned the Consent form were included in the study.

### 2.3 Data collection

The research team decided on key themes to discuss during the focus groups. The key themes included:

1. What extent was patient ethnicity considered when recruiting volunteer patients?
2. Current views on the representation of diverse patients during primary care placements and whether students receive sufficient clinical exposure to diverse patients to meet their learning needs.
3. Potential barriers/difficulties that arise when recruiting diverse volunteer patients.
4. Suggestions for improving the inclusion of diverse volunteer patients in primary care clinical placements.

Four focus group discussions with a total of 22 participants were conducted with GP tutors involved in recruiting volunteer patients (5–6 participants per group). We ensured diverse memberships in all focus groups. Focus group discussions lasted approximately 1 h. These focus groups were led by JS, an experienced qualitative researcher, and due to the COVID‐19 pandemic, were conducted virtually using Microsoft Teams, which enabled us to record the session for transcription. All the audio recordings were transcribed verbatim except all names of participants and places, which were anonymised.

### 2.4 Data analysis

We used Braun and Clarke's^17^ Thematic Analysis to analyse the narratives collected in the online focus groups. The initial stage of the analysis started with the familiarisation process, which involved reading and re‐reading transcripts.^18^ Focus groups were independently coded by JS and MM. Sections of the transcripts relevant to the research objects were highlighted and given shorthand labels or ‘codes’ to describe the content.^17^ Initial themes were generated by reviewing codes and identifying the similarities between them.^18^ The research team revised and refined the initial themes by assessing the data associated with each theme. Themes names were devised to capture the sense of each theme, and then the final themes were evidenced by data extracted directly from the transcripts.

### 2.5 Researcher reflexivity statement

Our aim was to be transparent about the research team and our roles in the project. Several of the authors were GPs, some Psychologists and one was a former student who had experienced teaching in General Practice. Some team members were from minority groups.

We recognised we had partial insider knowledge. We were open about this and tried to avoid confusion of our own experiences with those expressed by the study participants. Our varied backgrounds helped us contextualise the data. It also helped us to triangulate and ensure that we recorded participants' views accurately.

We tried to be open and honest about the purposes of the study and to take a non‐judgemental stance so that we could begin to consider how we can support practices in areas of low ethnic diversity to maximise opportunities for students.

## 3 RESULTS

From the analysis, we identified four key themes from the date: *Opportunistic Recruitment in Areas Lacking Diversity*, *Curriculum and Teaching Requirements Trumps Diversity of Volunteer*, *Diversity as a Barrier to Learning* and *Diversity as a Barrier to Recruitment*.

### 3.1 Theme 1: opportunistic recruitment in areas lacking diversity

#### 3.1.1 Recruitment driven by quantity not diversity of volunteers

For GP tutors, the recruitment of volunteer patients was just seen as something else that they had to do as part of their already demanding role, and therefore they were more interested in methods of recruitment that gave them the greatest number of suitable patients, as quickly as possible.

… and if you've got, you know, a certain number of names ticked off, you've got four patients for your eight students, you're kind of done aren't you, for that session (Focus Group 4)

This meant that there was no conscious consideration of the diversity of the patients they were recruiting as volunteers; they just wanted anyone who would agree (and was relevant to the teaching curriculum for that session). Once an adequate number of volunteer patients were recruited, no further efforts to recruit diverse volunteer patients were made.

… but is there a conscious effort? I don't necessary think so. I think it's kind of like fill the slot, get the bums on the chairs and then you kind of, her [teaching admin] job is done to some extent before she moves on to making sure the students are comfortable and things like that (Focus Group 4)

#### 3.1.2 Opportunistic recruitment does not equal diverse recruitment

For senior medical students, volunteer patients were often those patients who had contacted the practice to book an appointment. In these cases, GP tutors were openly driven by opportunity, recruiting patients who would have already been attending the practice, and the patient's background was never considered.

… they ring us for an appointment on the day because they have an acute problem, and then we offer if they would be happy to see a medical student with a GP tutor and so that teaching alone doesn't require any specific, you know, recognition of background. It's more based on medical presentation … (Focus Group 1)

In areas with low representation of, say, minority ethnic populations, these acute (opportunistic) patients are less likely to be ethnically diverse.

… it could also be because of where we are, we do have fewer minority patents in the surgery, so we tend to see more white patients, so I think that's where we tend to pick those ones that we see regularly or repeat (Focus Group 1)

### 3.2 Theme 2: curriculum and teaching requirements trumps diversity of volunteers

#### 3.2.1 Recruitment driven by clinical symptoms and diagnosis, not diversity

Considering the diversity of the patients they were recruiting as volunteers would only be another factor to consider when GP tutors said they were already facing many demands in terms of the medical curriculum and needing to deliver certain aspects within their teaching. Therefore, recruitment was largely driven by the clinical symptoms or diagnoses of the patients to facilitate student teaching.

When I'm recruiting patients, I'm thinking about the need like knee pain or back pain, and I'm not think about the ethnicity of the person (Focus Group 1)

Recruiting based on symptoms and diagnosis was time‐consuming enough, and in areas with low representation within the local area, the recruitment of ethnically diverse patients, for example, would make it an even more difficult task for GP tutors and would require more effort.

One of the real problems with primary care teaching is recruiting patients. It takes hours and hours. GPs spend hours on the phone trying to get people trying to get people who are relevant for modules. Their administrators and practice managers spend hours on the phone. They find the perfect patient who then isn't available on a Tuesday, and it really is a major piece of work (Focus Group 1)

Participants also acknowledged that the theme of recruiting based on clinical diagnosis and the pressure to get enough patients for teaching also exists when administrative staff recruit patients on behalf of the GP tutors.

So, we'll have a teaching admin and you kind of say, I need this many patients at this time and this many patients at this time, and these are the, these are the, um, diseases, pathological conditions that they need to have for the day (Focus Group 4)

He [teaching admin] sends texts to people, ask them if they'd be interested in seeing students. So, he sent the text, he wouldn't know their ethnicity. So sent the text to all the people with abdominal hysterectomies, and then the people who said, they'd be interested, then I just re I ring them all up. And it is quite, it's very difficult to get them (Focus Group 4)

#### 3.2.2 Neglecting diversity becomes usual practice

Participants acknowledged that they did not actively consider or neglected to think about diversity or representativeness when recruiting volunteer patients, and it could not be a priority for them.

I've never actually thought about it. I have never made a conscious effort to include people from non‐white backgrounds and recruit for teaching. I'm not sure why, maybe it is because it is [Location] and there's less representation here especially where we are in [Location], it is fairly sparce, that might have been a contributing factor but no, I can't say I've actually ever made a conscious effort to try and keep the recruitment process as broad as possible (Focus Group 1)

Importantly, there was a clear recognition that not only had the recruitment of patients from diverse backgrounds been forgotten, but also that non‐recruitment of patients from diverse backgrounds over time has become the 'norm’. They refer to the usual practice of getting anyone to volunteer for teaching and that ethnicity and other diversity characteristics were not actively considered.

After a bit of time, you just settled to it and that's your norm suddenly? It's very easy to get switched off about it, so you see what I mean? That it becomes the norm, that's what your practice is like (Focus Group 3)

### 3.3 Theme 3: diversity as a barrier to learning

#### 3.3.1 Additional communication needs seen to impair student learning

The GP tutors explained that students may find it more difficult to take a medical history from a patient if English is not their first language, without additional requirements such as interpretation services having to be made available. They were concerned that this could impair the students' learning, and thus they automatically discounted certain patients from being a volunteer patient. This directly impacted who was subsequently recruited. One GP tutor explained how this was directly linked to time.

Some of the reluctance is coming from me as well, in terms of recruiting and I just sometimes think, am I making it more difficult for the student if they've got a patient that they can't communicate as well with, in terms of taking a full history. And because we are pressed in terms of time, I think sometimes I do maybe subconsciously not recruit from other backgrounds (Focus Group 1)

Another talked about prioritising one particular facet of the history taking to aid student concentration:

… I have automatically discounted people who don't speak English well, as being unsuitable to speak to the students … and that can be whether somebody is hard of hearing, or whether they might be confused. We sort of try and choose easy patients for our first students … because we want them to just concentrate and get the structure of the history taking (Focus Group 3).

Participants also explained how they were more likely to choose cases where English was not the first language with their more experienced students as opposed to their first‐year students, who might find this more challenging.

I occasionally teach year 3 and we've got some year 4's who come to [Location] as well. And it wouldn't put me off, bringing patients in to see older or more experienced students, because I think it's a skill that they have to learn to be able to use language or cope with language difficulties (Focus Group 2)

#### 3.3.2 Fear of negative feedback from students

GP tutors wanted to ensure they avoided creating inadequate consultation and learning skills leading to negative feedback from their students. One said:

You do tend to anticipate almost negative feedback from the students, because they feel they haven't got the whole experience from that patient encounter and again that's something that I struggled against and that probably also affects my recruitment process (Focus Group 1)

However, this was not the same for all. One participant commented on how they actively encouraged the recruitment of volunteer patients with English as a second language to provide an opportunity for the students learning.

… I encourage them to have telephone triage, they do the telephone triage to start with, and I encourage them to do it with people who can't speak English. Because it then starts you off on that basic … and I just throw them into everything difficult … (Focus Group 1)

### 3.4 Theme 4: diversity as a barrier to recruitment

#### 3.4.1 Tutors' pre‐existing bias and assumptions

Cultural values and prior experiences may influence a tutor's (or recruiting staff's) views on whether patients' ethnicity might serve to mean they may be more/less inclined to participate as a volunteer patient, therefore affecting recruitment.

… they prefer to see the lady doctors if you're going to examine them down below, than if a male doctor would examine them, so I think it depends on the culture of … or even the doctors themselves, they still have their beliefs, they still have their cultures behind them. That doesn't just go away just because they are medical students or medical doctors (Focus Group 1)

Participants described, as part of their decision‐making process, that they have made a clear prior assumption that certain minority ethnic patients may feel less comfortable discussing certain medical topics (e.g. sexual health).

… because I teach obs and gynae [obstetrics and gynaecology] I would find maybe recruiting from BAME [Black, Asian, Minority Ethnic] backgrounds makes it more awkward so again they might struggle with some of the more sensitive questioning and I don't know whether that is just a bias that I've got, it might not be true but that's what stops me (Focus Group 1)

#### 3.4.2 Lack of familiarity affects diversity of recruitment

GP tutors acknowledged that they did not feel comfortable identifying certain patients based on their demographics and cold calling them for an appointment.

I think, unless you know people well, I think it's quite problematic. It feels uncomfortable to contact someone that you've never had clinical contact with and say, ‘I can see from our register that you know, you're in a same sex relationship, would you be prepared to volunteer, and come along and talk to the students about that’. I mean, what cold calling? What is that? You know, do you see what I mean (Focus Group 3)

One participant recognised that in view of the cultural differences and sensitivities, some ethnic minority patients may display a lack of ‘trust’ and require further explanation or counselling before allowing intimate examinations.

Well, I mean you really have to explain why you have to do it and they may not trust you in the beginning, but by the time you explain, they will feel a bit free to do that (Focus Group 1)

Therefore, they commented on how they would often use patients that they were familiar with or those who had been volunteer patients before, as these patients were more likely to agree to be a volunteer again, and it would be easier to arrange.

One of the things when it comes to inviting people in, I think it's about familiarity, knowing people and having created some sort of longitudinal relationship a lot of the time. I have very, very few people who are BAME (Focus Group 2)

## 4 DISCUSSION

This study set out to explore the inclusion of diverse volunteer patients in primary care clinical placements for medical student teaching. The study insights offer further explanation of the issues surrounding diverse patient representation within medical education and important clinical implications to the day‐to‐day work of medical doctors.

Our analysis shows that participants acknowledged the importance of ensuring that medical students have clinical experience in assessing and managing patients from diverse populations, but most did not actively think about the diversity of the patients they were recruiting.

Our work further developed the pre‐existing work of Forrest et al.,^12^ who looked at the diversity of medical education from the perspectives of heads of teaching. Tutors appear to prioritise the learning of specific subject material related to medical conditions over the skill of learning to communicate with patients from diverse groups. There is a need to develop clear and achievable learning outcomes, clarify perspectives on cultural diversity and ensure that cultural diversity is integrated across the entire curriculum.^19^

### 4.1 Strengths and limitations

To our knowledge, this study is the first of its kind to evaluate the barriers to recruiting diverse volunteer patients within primary care teaching from the perspective of GP tutors and that offers recommendations for improving and managing this. Indeed, it was notable that participants reported that taking part in the study in and of itself served to raise awareness of the current lack of diversity among volunteer patients.

In their guidance titled ‘Patient and Public Involvement in Undergraduate Medical Education’, the General Medical Council highlights the invaluable role of patients in medical teaching, including practising communicational skills, physical examinations and diagnostic skills.^20^ Furthermore, the *Royal College of General Practitioners* has published guidance on the principles of primary care teaching for medical students and described the usage of ‘selected patients’ for theme teaching.^21^ However, the precise model of patient recruitment likely varies between medical schools and there is little published data in their regard, thus, the findings may be limited in generalisability to all medical schools as there is little published data on the recruitment of volunteer patients for teaching undergraduate medical students for primary care placement. Nevertheless, it would be possible to generalise the findings to other medical schools and primary care contexts where voluntary patients are recruited as outlined in this paper for student teaching.

Moreover, we recognise that our study had a relatively small sample size due to difficulties recruiting GP tutors due to their busy schedules, especially as the study was conducted during the COVID‐19 pandemic. We initially intended to carry out these focus groups face‐to‐face during GP tutor development days/conferences where more GP tutors would be available to take part in focus groups, but because of the pandemic, this was not possible. Instead, focus groups had to be conducted online via Microsoft Teams, with the smaller sample of GP tutors we were able to recruit. Having said this, our study had a similar number of participants compared with other studies, including Forrest et al.,^12^ and we believe our findings add to the current body of research on this topic.

### 4.2 Implications for research and/or practice

The NHS Long Term Workforce Plan 2023^22^ supports diversity in NHS entrant recruitment to reflect the communities served in order to reduce inequalities. Reflecting on both our experience and the experience of other GP tutors expressed in this study, breaking barriers to diversify patient recruitment involves consideration of active enrolment of ethnic minority patients for teaching. This may need to be considered at the university level to consider the development of a policy to provide guidelines for this if required.

Clinically, patients are recruited for teaching via diagnosis codes in their records. Ethnicity is not routinely coded into records and therefore cannot be ‘searched’ for. Given that patients with protected characteristics have reduced access to and engagement with healthcare,^23^ then it can be reasoned that there will be lower numbers of diverse patients found through diagnosis‐driven searches. GP tutors in practices may need to consider prospective (and active) recruitment of diverse patients for medical students.

This study identified various barriers that limit the recruitment of diverse volunteer patients for medical student teaching, and our findings have allowed us to develop a series of recommendations for ways of improving diversity among volunteer patients (see Table 1).

**TABLE 1:** Recommendations to increase the recruitment of diverse patients for medical education.

Raise awareness of diverse representation in primary care teaching, making explicit links to learning outcomes (i.e. that it is a requirement in the medical curriculum).
GP tutors should be made aware of the importance of diverse representation in primary care and student teaching. This should be explicitly linked to learning outcomes, medical school curriculum, and the requirement that students gain clinical experience working with patients from diverse groups so that they are more adequately prepared to deliver optimal clinical care to all patients once they are qualified. This could also be included in GP tutor training days, handbooks and so forth.
Interpretation services for voluntary patients
For student learning, it is important that they have practice dealing with patients whose first language is not English. Interpretation services should be made available so these patients are able to volunteer and students are able to communicate with them effectively. GP tutors will also not have to worry about student experience and negative student feedback, as students will be able to have an adequate consultation with the patient and experience using these services. Interestingly, the use of interpretation services has not been incorporated into even postgraduate primary care assessments as of date.
Rotating students around practices
Demographic information on the diversity of patients should be collected and stored at each practice. By knowing this information, students could then be rotated around practices so that they have the opportunity to gain clinical experience with patients from diverse backgrounds.
Use opportunities to teach students about diversity at any time
If there are issues with low recruitment of diverse patients at a practice, GP tutors should use other opportunities to teach students about the clinical relevance of diversity among patients and how it can influence a patient's presentation, attitudes towards healthcare, and beliefs surrounding treatments and complications, which may be more common in certain patient groups. They do not need to have a diverse patient present; instead, GP tutors, for example, could explain to students how a condition manifests differently (or the same) in a more diverse patient or incorporate diversity with anonymised patient case presentations.
Allow more time for the recruitment of diverse patients
Recruiting volunteer patients is already time‐consuming for GP tutors, and the recruitment of diverse patients can require even more effort (e.g. searching for ethnically diverse patients in databases or arranging translators for patients whose first language is not English). GP tutors should be given the time to put in the effort to recruit more diverse patients for student teaching.
Give diverse patients and students the option to first start with telephone triage.
Work differently across different years of study
Give diverse patients and students the option to first start with telephone triage. Bringing in diversity in different ways depending on the confidence and experience of the students. For example, first‐year students may find it more difficult to have a consultation with a patient whose first language is not English, and this may be something more beneficial to do with students with more experience.
Use online educational materials to supplement
Online educational materials could be used to supplement teaching and learning. For example, in areas where ethnic diversity is low, GP tutors can use online material to supplement teaching (e.g. skin colour presentation).
Acknowledge patient participation
Financial rewards are not always needed. Patients are often happy to get feedback about how their time has been helpful for students and other patients. Say thank you to patients and acknowledge those who may have gone out of their comfort zone. Acknowledge, respect and validate different health care beliefs. Give feedback to patients about how important it is to listen to individual views and experiences of healthcare.
Increase awareness to patients
Explain to patients what you are doing and why it is important to involve them. To aid the recruitment, GP tutors could be provided with patient recruitment resources in several languages.
Make an effort to keep a record of patient demographics (e.g. ethnicity)
Demographic information on the diversity of patients should be collected and stored at each practice with their consent. Some GPs may find it difficult to ask patients about their ethnicity, or sexual orientation, for example, in seeking diverse representation, but it is important to reassure them that they just need to be open about the reasons for asking.
Send GP tutors reminder messages to consider diversity when recruiting patients
Reminder messages should be sent to GP tutors to remind them to consider the diversity of the patients that they are recruiting throughout the year to ensure that there is enough diversity and students get experience with diverse patient groups.
Incorporate ethnicity when conducting patient searchers on clinical databases
When conducting database searches for volunteer patients, effort should be made to consider diversity. An effort should be made to collect demographic information from patients to ensure that this process can be done.
Create a list of potential patients when seeing them in practice
During day‐to‐day routine practice, GP tutors should identify patients who would be suitable for student teaching. We encourage GP tutors to create a list of potential patients (with diversity considered where possible), which they can easily refer to when recruiting volunteer patients. This would hopefully result in a less time‐consuming process when they need to recruit volunteer patients.

### 4.3 Future research

Additional research could be conducted to study this issue in other primary care settings, secondary care settings or from the perspective of diverse patients. As this study was only conducted in the Norfolk area in the United Kingdom, this could be developed into a multi‐centre study that could include other practices that are experiencing issues in recruiting other protected characteristics as well as ethnicity. It is not just GP tutors that are involved in the recruitment of volunteer patients, but the administrative staff at the surgeries too. Therefore, future research could also include the administrative staff who are involved in the recruitment of volunteer patients.

## 5 CONCLUSIONS

As the General Medical Council^1^ states, medical school curricula must give medical students experience with the diverse patient groups that they would see working as a doctor once they are qualified. GP tutors need to make conscious effort when it comes to recruiting diverse patient volunteers. Having experience with diverse patient groups has shown to increase students' self‐perceived preparedness to assess and manage these patient groups.^24^ Patient populations will continue to become more diverse, and therefore, medical schools must prepare their students.

## AUTHOR CONTRIBUTIONS

**Mohammad Malik:** Conceptualization; investigation; formal analysis; writing—review and editing. **Leanne Tyson:** Writing—original draft; writing—review and editing; formal analysis. **Pauline Bryant:** Conceptualization; writing—review and editing; investigation; supervision. **Payal Patel:** Investigation; writing—review and editing. **Richard Young:** Investigation; writing—review and editing; methodology. **Joanna Semlyen:** Conceptualization; investigation; writing—original draft; writing—review and editing; methodology; formal analysis; supervision; data curation; project administration; resources; validation.

## CONFLICT OF INTEREST STATEMENT

The authors declare no competing interests.

## ETHICAL APPROVAL

This study was approved by the University of East Anglia Faculty of Medicine and Health Sciences Research Ethics Committee (Reference: ETH201/22/112).

## Data Availability

The data that support the findings of this study are available from the corresponding author upon reasonable request.
